# Comparison of Weightlifting, Traditional Resistance Training and Plyometrics on Strength, Power and Speed: A Systematic Review with Meta-Analysis

**DOI:** 10.1007/s40279-021-01627-2

**Published:** 2022-01-13

**Authors:** Stephanie J. Morris, Jon L. Oliver, Jason S. Pedley, G. Gregory Haff, Rhodri S. Lloyd

**Affiliations:** 1 Youth Physical Development Centre, Cardiff School of Sport and Health Sciences, Cardiff Metropolitan University, Cardiff, UK; 2 Sport Performance Research Institute, New Zealand (SPRINZ), AUT University, Auckland, New Zealand; 3 School of Medical and Health Sciences, Edith Cowan University, Joondalup, WA Australia; 4 Centre for Sport Science and Human Performance, Waikato Institute of Technology, Hamilton, New Zealand; 5 Directorate of Psychology and Sport, University of Salford, Salford, Greater Manchester M6 6PU UK

## Abstract

**Background:**

Weightlifting training (WLT) is commonly used to improve strength, power and speed in athletes. However, to date, WLT studies have either not compared training effects against those of other training methods, or been limited by small sample sizes, which are issues that can be resolved by pooling studies in a meta-analysis. Therefore, the objective of this systematic review with meta-analysis was to evaluate the effects of WLT compared with traditional resistance training (TRT), plyometric training (PLYO) and/or control (CON) on strength, power and speed.

**Methods:**

The systematic review included peer-reviewed articles that employed a WLT intervention, a comparison group (i.e. TRT, PLYO, CON), and a measure of strength, power and/or speed. Means and standard deviations of outcomes were converted to Hedges’ *g* effect sizes using an inverse variance random-effects model to generate a weighted mean effect size (ES).

**Results:**

Sixteen studies were included in the analysis, comprising 427 participants. Data indicated that when compared with TRT, WLT resulted in greater improvements in weightlifting load lifted (4 studies, *p* = 0.02, *g* = 1.35; 95% CI 0.20–2.51) and countermovement jump (CMJ) height (9 studies, *p* = 0.00, *g* = 0.95; 95% CI 0.04–1.87). There was also a large effect in terms of linear sprint speed (4 studies, *p* = 0.13, *g* = 1.04; 95% CI − 0.03 to 2.39) and change of direction speed (CODS) (2 studies, *p* = 0.36, *g* = 1.21; 95% CI − 1.41 to 3.83); however, this was not significant. Interpretation of these findings should acknowledge the high heterogeneity across the included studies and potential risk of bias. WLT and PLYO resulted in similar improvements in speed, power and strength as demonstrated by negligible to moderate, non-significant effects in favour of WLT for improvements in linear sprint speed (4 studies, *p* = 0.35, *g* = 0.20; 95% CI − 0.23 to 0.63), CODS (3 studies, *p* = 0.52, *g* = 0.17; 95% CI − 0.35 to 0.68), CMJ (6 studies, *p* = 0.09, *g* = 0.31; 95% CI − 0.05 to 0.67), squat jump performance (5 studies, *p* = 0.08, *g* = 0.34; 95% CI − 0.04 to 0.73) and strength (4 studies, *p* = 0.20, *g* = 0.69; 95% CI − 0.37 to 1.75).

**Conclusion:**

Overall, these findings support the notion that if the training goal is to improve strength, power and speed, supplementary weightlifting training may be advantageous for athletic development. Whilst WLT and PLYO may result in similar improvements, WLT can elicit additional benefits above that of TRT, resulting in greater improvements in weightlifting and jumping performance.

**Supplementary Information:**

The online version contains supplementary material available at 10.1007/s40279-021-01627-2.

## Key Points

**Table:** 

Weightlifting training and plyometric training may result in similar improvements in strength, power and speed
Weightlifting training may elicit additional benefits above that of traditional resistance training, resulting in greater improvements in weightlifting and countermovement jump performance
Future research should investigate the means by which weightlifting training, plyometric training and traditional resistance training can be effectively combined in a periodized plan

## Introduction

Weightlifting is a competitive sport that requires athletes to lift a maximal amount of weight in the snatch and clean and jerk. In competition three attempts are made with each lift and the maximal weight lifted for each lift is summed to determine a winner [1]. Weightlifting exercises and their derivatives (e.g. hang clean, hang snatch, power clean, power snatch, high pull) have become a popular training modality to improve physical attributes underpinning performance across a range of sports [2–4], largely owing to the high strength and power expressions during the movements [5]. The magnitude of force production and the capacity to perform a given amount of work as rapidly as possible are often suggested as primary underpinning qualities of sport skills such as jumping, sprinting and change of direction tasks [6–10]. Therefore, developing strength, power and speed capabilities are often primary aims of many athletic development programmes. Furthermore, since reduced muscular strength, greater strength imbalances and slow sprint speeds are associated with increased musculoskeletal injury risk [11, 12], improvements in strength, power and speed are often desirable to help mitigate injury risk.

Existing meta-analyses examining the effects of weightlifting training (WLT) on jump performance advocate for this type of training as an effective training mode to improve vertical jump performance [13, 14], which is most often determined by jump height. Several researchers have highlighted strong relationships between load and movement velocity, with the assessment of strength qualities being load-velocity specific [15, 16]. Therefore, the assessment of jump performance provides only a measure of force production or strength qualities under low load and high velocity demands. The high power outputs and rate of force development expressed in weightlifting movements [17], in conjunction with the motor control and coordination demands on the trunk and lower body muscles to stabilise and transmit forces [18], can effectively impact various aspects of an athlete’s load-velocity profile and facilitate the development of a range of physical qualities across the strength and power continuum [19, 20]. However, extant meta-analyses have solely focused on the effects of WLT on jump performance alone, with no meta-analyses providing comprehensive estimates of the effect of WLT on measures of strength, power and speed. Thus, the pooled effects of WLT on physical performance across the spectrum of load-velocity demands remains unclear.

Various forms of strength and power training have been shown to improve measures of strength, power, change of direction speed (CODS) and linear sprint speed [21–24]. Resistance training is a collective term that refers to methods of physical conditioning that involve the progressive use of a wide range of resistive loads, different movement velocities and a variety of training modalities [25]. Whilst resistance training has previously been shown to be effective for improving muscle strength and power [26–28], improvements in speed performance may be enhanced when resistance training is performed in a mixed method approach (i.e. concurrent with weightlifting exercises), rather than a traditional resistance training (TRT) method approach (resistance training alone) [29–31]. When comparing the impact of WLT and TRT on power generation capacity the findings are equivocal, with research in favour of both TRT [32] and WLT [33, 34]. Plyometric training (PLYO) consists of quick, powerful actions that involve muscle lengthening immediately followed by rapid shortening of the same muscle [25]. Examples of plyometric exercises include explosive jumps, hops, bounds, and skips. Possibly owing to the demand for higher force production at higher velocities, WLT and PLYO have been shown to exhibit a modest advantage over TRT for improvements in power and speed measures [34, 35]. When comparing improvements in strength from WLT and PLYO, findings from Moore et al. [36] suggest the training methods result in similar strength gains, whilst findings from Tricoli et al. [37] suggest PLYO may be superior. Despite these findings, there is no reported consensus highlighting the magnitude of differences between WLT and other strength and power training methods on measures of strength, power and speed. Therefore, the purpose of this meta-analysis was to investigate whether WLT resulted in greater improvements in strength, power and speed compared with TRT, PLYO and control groups that did not complete any training (CON). It was hypothesised that WLT and PLYO may elicit adaptations in a wider range of physical qualities across the strength and power continuum in comparison with TRT. Furthermore, a secondary goal was to establish practical applications and guidelines for researchers and practitioners employing and investigating these training methods.

## Methods

The meta-analysis was conducted in accordance with the 2020 Preferred Reporting Items for Systematic Reviews and Meta-Analyses (PRISMA) guidelines [38]. Consultation of Prospero indicated that the review did not need to be registered because no health-related outcomes were measured.

### Eligibility Criteria

In line with the Population, Intervention, Comparison and Outcomes (PICO) framework, for eligibility in the review, studies must have conducted a WLT intervention; attained pre- and post-training measurements in strength, power, speed or CODS outcome measures; and included either an appropriate comparison training group who performed either TRT or PLYO, or a CON group. To be deemed an appropriate WLT intervention, the intervention must have included more than one weightlifting exercise within the training session. Since weightlifting exercises are very rarely used in isolation and WLT interventions regularly include accessory strength exercises (e.g. squats, deadlifts), it was deemed appropriate to group weightlifting interventions comprising solely weightlifting exercises and weightlifting exercises with supplementary strength exercises together. The WLT interventions must have prescribed more than one weightlifting exercise per week and a minimum of one weightlifting exercise per training session; however, no intervention duration exclusions were applied. A CON group was defined as a group that performed no additional training beyond sports practice or typical physical activities. Where studies included a CON group that still participated in TRT [39, 40], these were instead categorised as TRT groups. TRT was defined as an intervention that involved the progressive use of a wide range of resistive loads, different movement velocities and training modalities (e.g. free-weight exercises using barbells, dumbbells and kettlebells), while PLYO was defined as a form of training involving body weight jumps, hops, bounds, and/or skips. There were no limitations on study population, participant age, maturity or sex. Further exclusion criteria included non-English language publications, abstract-only articles and insufficient information about the WLT intervention (with detail on training frequency serving as a minimum requirement).

### Information Sources and Search Strategy

To obtain relevant literature on WLT interventions, four electronic databases were searched on April 5, 2021: MEDLINE (via Ovid), SPORTDiscus (EBSCOhost), PubMed, and SCOPUS. Candidate search terms were identified by screening titles, abstracts and subject indexing of known, relevant studies. Using these terms, a pilot search was performed to identify the need for any additional terms. The following Boolean search syntax were used: ((olympic OR snatch* OR power clean* OR hang clean* OR clean and jerk OR jerk* OR high pull* OR weightlift*) AND (training or intervention)) to search title and/or abstract and/or keywords of articles. Search terms for each database are presented in Appendix 1 (see electronic supplementary material [ESM]). Searches were limited to journal sources, excluding publications from a dissertation, thesis, magazine article, or from a non-peer reviewed source. There was no search limitation for publishing date. The reference list of each included study was screened by title to identify any additional suitable studies for inclusion in the review.

### Study Records

From the initial search, study titles and abstracts were screened by a single reviewer [41] to remove duplicates, non-English language publications, non-empirical research (e.g. reviews, meta-analyses, commentaries and letters), research without a comparative repeated measures design (e.g. cross-sectional studies, single-group studies and case studies) and clearly irrelevant studies (e.g. studies that did not include a WLT intervention). The full texts of the remaining articles were reviewed for final inclusion based on the following criteria: (i) a full text of an article was available, excluding abstract-only articles; (ii) the study employed a WLT intervention inclusive of more than one weightlifting exercise per week and a minimum of one weightlifting exercise per training session; (iii) the study included an appropriate comparison group comprising a TRT, PLYO or CON group; (iv) the study reported pre- and post-training measurements in a strength, power, speed or CODS assessment; (v) sufficient information about the WLT intervention was included, with detail on training frequency serving as a minimum requirement.

### Data Items

The following data were extracted from the articles: (i) sample size; (ii) participant characteristics (age, sex, sport, training experience); (iii) intervention duration; (iv) intervention prescription (training frequency, exercises prescribed, sets, repetitions, intensity, rest); (v) reported variables from the strength, power, speed or CODS testing and (vi) means and standard deviation (SD) for the pre- and post-intervention testing data. Categorisation of the strength and power assessments, including the specific tests and outcome measures, are presented in Appendix 2 (ESM). Where multiple performance variables were collected for a single test within a study, the most common test and metric across the included studies was extracted and reported. In instances where insufficient information was available for mean and SD data extraction, lead authors were contacted and asked to provide the data. In instances where no response was received, the study was excluded. Where test results were duplicated across studies, data were extracted from the most comprehensive report only. All study exclusion and data extraction was verified by a second reviewer to minimise potential selection bias and data extraction errors [42]. In the event of disagreement, a decision was reached by a vote, inclusive of a third reviewer.

### Risk of Bias Assessment

The Tool for the assEssment of Study qualiTy and reporting in EXercise (TESTEX) Scale (presented in Appendix 3, ESM) was used to assess the methodological quality of the included studies as this is considered a reliable and valid tool to report on the methodological quality in exercise training studies [43]. Each item on the TESTEX checklist is answered with ‘yes’ if the criteria are satisfied and associated with a point, or with a ‘no’ if the criteria are not satisfied. Items 6 and 8 have three and two questions and therefore, three and two associated points, respectively. The maximum number of possible points on the checklist is 15. Based on the summary scores, study quality was classified as ‘excellent’ (12–15 points), ‘good’ (9–11 points), ‘fair’ (6–8 points), or ‘poor’ (< 6 points) [44]. Studies were rated independently by two reviewers and Cohen's kappa was calculated to assess the measurement agreement between the two raters. In the event of disagreement, a decision was reached by vote, inclusive of a third reviewer. Any studies scoring as ‘poor’ methodological quality (TESTEX score < 6) were excluded from the analysis.

In order to examine for potential publication bias, a post-hoc risk-of-bias-related sensitivity analysis was conducted, removing all fair quality (score 6–8 on the TESTEX scale) studies for all main outcome parameters. In addition, an empirical funnel plot evaluation was performed, observing the symmetry and inverted funnel shape appearance of the plots. Statistical tests for detecting funnel-plot asymmetry such as Begg’s rank correlation test and Egger’s linear regression test were not used in this analysis due to their low statistical power [45].

### Data Synthesis

To allow comparison between the outcome measures of the selected studies, effect sizes (ES) with 95% confidence intervals (CI) were calculated. Effect sizes (Hedges’ *g*) were calculated from the difference between the standardised mean change for the WLT and respective comparison group, divided by the pooled and weighted estimates of SD [46]. To account for the positive bias associated with small samples, a correction factor was applied [46]. The studies included in the review were drawn from different populations, included different training intervention prescriptions and utilised a variety of strength and power assessments and variables; all factors that may have influenced the training effect. Therefore, the random-effects model was used to conduct the meta-analysis [47], using the DerSimonian and Laird inverse variance method [48]. The Review Manager computer software (RevMan; Version 5.4.; Copenhagen: The Nordic Cochrane Centre, The Cochrane Collaboration, 2014) was used to conduct the analysis. If there were found to be less than two studies reporting a strength, power, speed or CODS test within the comparison groups, the data were not reported in the meta-analysis. Forest plots with 95% CI were created and ES were classified according to the following scale: 0–0.19 = negligible effect, 0.20–0.49 = small effect, 0.50–0.79 = moderate effect and ≥ 0.80 = large effect [49]. Effects were considered statistically significant at *p* < 0.05 and approaching significant when 0.05 < *p* < 0.1.

The chi-square test (*χ*^2^) was used to determine if statistical heterogeneity was present. To compensate for the low power of the chi-square test when few studies are included, heterogeneity was tested at an alpha level of *p* < 0.10 rather than at *p* < 0.05 [50, 51]. To quantify the percentage of variation across studies due to heterogeneity, rather than chance, the *I*^2^ statistic was used together with the observed effects. *I*^2^ values of 25%, 50% and 75% were interpreted as representing small, moderate and high levels of heterogeneity [50]. The importance of the observed *I*^2^ value was interpreted in relation to the magnitude and direction of effects and strength of evidence for heterogeneity. In addition, when more than two studies were included in the comparison, prediction intervals were calculated [52] to provide an index of dispersion and information on how widely the effects vary.

## Results

### Study Characteristics

**Fig. 1:**
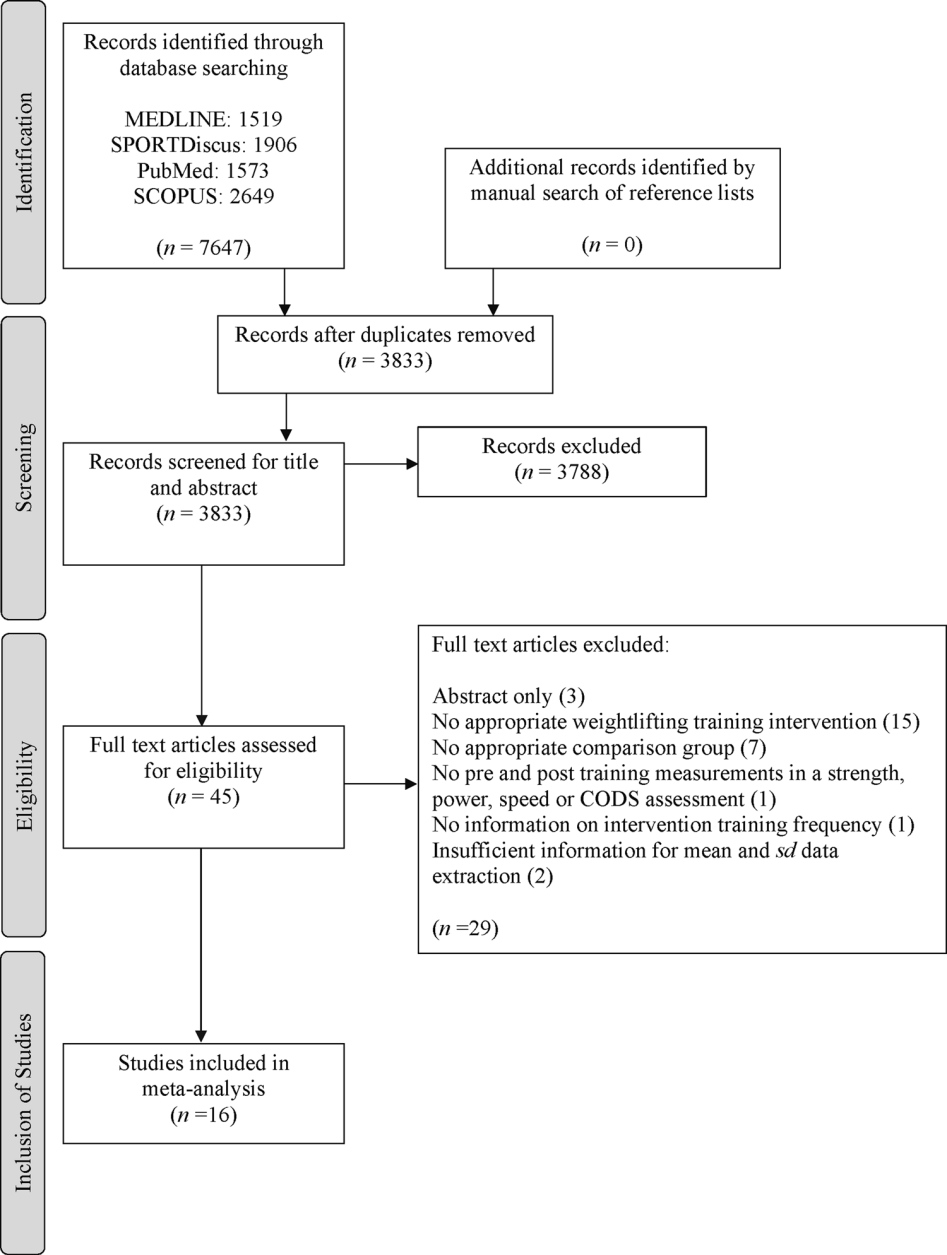
Summary flowchart of literature search, screening process and outcome

The study selection processes and search findings are presented in Fig. 1. The online database search returned 7647 results, once duplicates between the database results were removed, 3833 articles remained. The preliminary search of the titles and abstracts removed a further 3788 articles due to the pre-determined inclusion criteria. From the remaining studies (*n* = 45), no additional articles were identified from the screen for relevant missed articles. Full texts were reviewed and a further 29 manuscripts were removed due to one of the exclusion criteria. Two studies met the study inclusion criteria, however were removed due to insufficient information for data extraction and no author response [35, 53]. Following all screening processes, a total of 16 studies comprising 427 participants met the inclusion criteria and were used for the meta-analysis (Fig. 1). Of these 16 studies, five included a CON group with a total sample size of 53 participants [34, 37, 54–56], 10 included a TRT group with a total sample size of 127 participants [32–34, 39, 40, 54, 57–60] and six included a PLYO group with a total sample size of 61 participants [36, 37, 56, 60–62]. The total sample size of the WLT groups was 186 participants.

### Risk of Bias

A summary of the methodological assessment for all studies included in the review is shown in Table 1. There was 95.3% agreement (κ = 0.919; *p* < 0.001) between the two reviewers with nine instances of disagreement. Of the nine disagreements, eight were resolved through discussion between the reviewers, whilst a third reviewer was required to resolve the one remaining disagreement. The median total score for the included studies was 9 (range 6–12) out of the 15 possible points, suggesting the findings from the meta-analysis are based on good quality research. As all studies were of ‘fair’ methodological quality or above (TESTEX score ≥ 6), no study was excluded from the review on the basis of the screening outcome. Studies scored highly for reporting of point measures and measures of variability for outcome measures (*n* = 16), reporting of intervention programme prescription (e.g. volume; *n* = 16), reporting of between-group statistical comparisons (*n* = 15) and appropriate intervention prescription to ensure exercise load is titrated to keep relative intensity constant or progressive (*n* = 14). In contrast, all studies failed to include activity monitoring in the comparison groups (*n* = 16) and a large majority of studies failed to meet criteria such as blinding of assessor for at least one key outcome (*n* = 14) and allocation concealment (*n* = 13).

**Table 1:** Outcomes of TESTEX methodological screening tool performed on included studies

Study	Study quality					Study reporting							Total/15
	Item 1	Item 2	Item 3	Item 4	Item 5	Item 6	Item 7	Item 8	Item 9	Item 10	Item 11	Item 12	
Arabatzi and Kellis [54]	1	1	0	1	0	0	1	2	1	0	1	1	9
Helland et al. [32]	1	1	1	1	0	1	0	2	1	0	1	1	10
Arabatzi et al. [61]	1	1	0	0	0	0	1	2	1	0	1	1	8
Hermassi et al. [40]	0	1	0	0	0	0	1	2	1	0	1	1	7
Oranchuk et al. [57]	1	1	0	0	0	2	1	2	1	0	1	1	10
Teo et al. [62]	1	1	1	0	0	2	1	2	1	0	1	1	11
Hoffman et al. [33]	0	0	0	0	0	3	0	2	1	0	1	1	8
Otto et al. [58]	1	1	0	0	0	0	1	2	1	0	0	1	7
İnce [55]	0	1	0	0	0	0	0	2	1	0	1	1	6
Channell and Barfield [34]	1	0	0	1	0	0	1	1	1	0	1	1	7
Hermassi et al. [39]	0	1	1	1	1	2	1	2	1	0	1	1	12
Pichardo et al. [59]	1	1	0	1	0	1	0	2	1	0	1	1	9
Hawkins et al. [60]	1	1	0	1	1	0	1	2	1	0	0	1	9
Moore et al. [36]	1	0	0	0	0	1	1	2	1	0	1	1	8
Tricoli et al. [37]	1	1	0	0	0	3	0	2	1	0	1	1	10
Kaabi et al. [56]	1	1	0	1	0	0	1	2	1	0	1	1	9

Repeating the meta-analysis after removing the studies of fair quality (6–8 points) for the sensitivity analysis did not materially change the results for the main outcome parameters in the WLT versus CON and WLT versus PLYO analysis. However, in the WLT versus TRT comparison, removing the studies of fair quality resulted in negligible and small, non-significant ES in favour of TRT for improvements in squat jump (SJ) and strength performance, respectively (Appendix 5 in the ESM). The funnel plot evaluation (presented in Appendix 4, ESM) showed no obvious risk of bias in WLT versus CON. In the WLT versus TRT and WLT versus PLYO comparisons, an overrepresentation of larger studies is apparent, as evidenced by a deficit at the bottom of the funnels. The gap on the lower left-hand side of the plots suggests a lack of small studies with negative results. The ‘trim and fill’ method [63] was not used to identify and correct the funnel plot asymmetry due to its poor performance in the presence of substantial between-study heterogeneity [64, 65].

### Description of Studies

#### Participant Characteristics

Table 2 provides a summary of the included studies. The median WLT group size across all the studies was 11 participants (range 7–31). In the studies comparing WLT and CON, median WLT group size was 11 participants (range 7–17), while the median CON group size was 8 participants (range 6–17). In the studies comparing WLT and TRT, median WLT and TRT group size was 11 (range 9–31) and 10 participants (range 9–28), respectively, and in the studies comparing WLT and PLYO, the median number of individuals in the WLT and PLYO groups was 9 (range 7–15) and 10 participants (range 7–15), respectively.

**Table 2:** Summary of studies investigating the effects of weightlifting interventions

Study	Participant characteristics			Test(s)	Variable(s)	Weightlifting intervention	
	Sample size (*n*)	Age group (mean years ± SD)	Demographics			Duration	Training frequency, exercises prescribed, sets, repetitions, intensity, rest)
*Weightlifting training and control group comparisons (n = 5)*							
Arabatzi and Kellis [54]	26, WLT group (9), TRT group (9), CON group (8)	20.3 ± 2.0	Male, students of physical education in Greece	SJ	Jump height (cm) Hip and knee displacement	8 weeks	WLT group: 3 × /week; snatch from a squat position, high-pull, power clean, half-squat, and clean and jerk; 4 sets of 4–6 reps; 75–90% 1RM, 3-min rest. TRT group: 3 × /week; knee extension, knee flexion, push, pull of biceps femoris, and half-squat; 4 sets of 4–6 reps; 75–90% 1RM; 3-min rest. CON group: standard sport activities through academic programme, no additional weight exercises
				CMJ	Peak power (W)		
				DJ (20, 40, 60 cm)	Jump height (cm)		
Channell and Barfield [34]	27, WLT group (11), TRT group (10), CON group (6)	15.9 ± 1.2	Male, student athletes, from the high school football programme, limited resistance training experience	Power clean 1RM Back squat 1RM	Weight (kg)	8 weeks (prior 4 weeks of TRT only)	WLT group: 3×/week; power clean, hang clean, snatch pulls, snatch, push jerk and accessory strength; 3–5 sets of 5–10 reps; 60–75% 1RM. TRT group: 3×/week; bench press, squat, dead lift, leg press, upper body press, back extensions, abdominals; 3–5 sets of 5–20 reps; 60–100% 1RM. CON group: did not participate in any off-season training
				CMJ	Height (cm)		
Tricoli et al. [37]	21, WLT group (7), PLYO group (7), CON group (7)	22.0 ± 1.5	Male, limited WLT experience	½ Back squat 1RM SJ CMJ	Weight (kg) Height (cm)	8 weeks	WLT group: 3×/week; WL full exercises, WL derivatives and accessory strength; 3–4 sets, 4–6 reps; 80–90% 1RM. PLYO group: 3×/week; plyometrics, half-squat; 4–10 reps, 4–6 reps; 80–90% 1RM. CON group: no training
				10, 30-m sprint Agility	Time (s)		
İnce [55]	34, WLT group (17), CON group (17)	15.4 ± 1.6	Female, volleyball players, 3 years volleyball experience	CMJ	Jump height (cm) Stiffness (kNm)	6 weeks	WLT group: 2×/week, Olympic split lifts, hang split snatch and clean and jerk in addition to normal volleyball training; 3–7 sets of 5 reps; 70–90% 1RM; 2 min. CON group: normal volleyball/ technical training only
				5, 20-m sprint T-test (CODS)	Time (s)		
Kaabi et al. [56]	45, WLT group (15), PLYO group (15), CON group (15)	16.6 ± 0.4	Male, ‘recreationally trained’ resistance training experience	Squat 1RM SJ CMJ	Height (cm)	8 weeks	5×/week table tennis technical sessions in addition to WL or PLYO. WLT group: 2×/week; WL derivatives and accessory strength; 3–4 sets, 5–10 reps; 70–85% 1RM; 3–5 min rest. PLYO GROUP: 2×/week; plyometrics; 3–4 sets, 5–10 reps; 70–85% 1RM; 3–5 min rest. CON group: 5× table tennis technical sessions/week with no TRT
				5-m sprint *T* test (CODS)	Time (s)		
*Weightlifting training and traditional resistance training comparisons (n = 10)*							
Arabatzi and Kellis [54]	26, WLT group (9), TRT group (9), CON group (8)	20.3 ± 2.0	Male, students of physical education in Greece	SJ	Jump height (cm) Hip and knee displacement	8 weeks	WLT group: 3×/week; snatch from a squat position, high-pull, power clean, half-squat, and clean and jerk; 4 sets of 4–6 reps; 75–90% 1RM, 3-min rest. TRT group: 3×/week; knee extension, knee flexion, push, pull of biceps femoris, and half-squat; 4 sets of 4–6 reps; 75–90% 1RM; 3-min rest. CON group: standard sport activities through academic programme, no additional weight exercises
				CMJ DJ (20, 40, 60 cm)	Peak power (W) Jump height (cm)		
Helland et al. [32]	26, WLT group (13), free weight/TRT group (13)	20.0 ± 3.0	Male (*n* = 29) and female (*n* = 10), Badminton, volleyball, and hockey players, Norwegian high school for elite sports, limited WLT experience	Back squat 1RM SJ CMJ	Weight (kg) Jump height (cm)	8 weeks	WLT group: 3×/week; 45 min; 0full cleans with front squat, hang cleans, power jerk behind the neck, full snatches, and hang snatches; 2–5 sets of 3–5 reps; 85–95% 1RM; 3-min rest. Free weight/TRT group: 3×/week, 45 min; squat, single leg squat, CMJ; 2–6 sets of 3–5 reps; 10–93% 1RM; 3-min rest
				Loaded CMJ (10–80 kg) DJ (40 cm) 30-m sprint	Peak power (W) Jump height (cm) Time (s)		
Hermassi et al. [40]	22, WLT group (11), CON group (11)	20.7 ± 0.5	Male, handball players, playing experience 9.2 ± 0.7 years	Snatch 1RM C&J 1RM Back half-squat 1RM	Weight (kg)	12 weeks	WLT group: In addition to CON group training, 2× WLT/week to replace technical-tactical sessions; snatch from a squatting position, bench press, half-squat, and clean and jerk; 1–3 sets of 8–12 repetitions; 55–75% 1RM; 3-min rest. CON group: 6×/week typical handball training programme inclusive of resistance training
				SJ	Jump height (cm)		
				CMJ	Relative peak power (W/kg)		
				5, 30-m sprint	Time (s)		
				T-half test (CODS) Handball throwing	Velocity (m/s)		
Oranchuk et al. [57]	18, hang pull/WLT group (9), jump squat/TRT group (9)	Male 19.6 ± 2.7, female 21.4 ± 3.0	Male (*n* = 8) and female (*n* = 10), swimmers, minimum of 1-year resistance training experience	SJ CMJ IMTP	Jump height (cm) Relative peak power (w/kg) Relative peak force (w/kg) Peak RFD (N/s) Relative force at 50–250 ms time bands (N/kg)	10 weeks	WLT group: 2×/week; high hang pulls, squat, deadlift, bench press, rows, pull ups and lunges; 2–6 sets of 2–12 reps; 60–90% 1RM power clean; 2–3-min rest. Jump squat/TRT group: trap-bar jump squat, squat, deadlift, bench press, rows, pull ups and lunges; 2–6 sets of 2–12 reps; 10–80% 1RM; 2–3 min
Hoffman et al. [33]	20, WLT group (10), TRT group (10)	WLT group 19.3 ± 1.2, power lifting group 18.9 ± 1.4	Male, NCAA Division III football team	Back squat 1RMs	Weight (kg)	15 weeks (first 6 weeks general strength only)	WLT group: 4×/week; additional 2×/week speed and agility training; WL full lifts, WL derivatives and accessory strength; 2–5 sets of 3–10 reps; 80–95% 1RM. TRT group: 4×/week; additional 2×/week speed and agility training; squats, deadlifts, upper body pushing and pulling, calf raises, sit ups; 3–5 sets of 4–10 reps; 80–95% 1RM
				CMJ	Jump height (cm)		
				40-yard sprint T drill (CODS)	Time (s)		
Otto et al. [58]	30, WLT group (13), kettlebell/TRT group (17)	WLT group 22.9 ± 2.0 kettlebell group 22.8 ± 1.9	Male, minimum of 1-year resistance training experience	Power clean 1RM Back squat 1RM	Weight (kg)	6 weeks	WLT group: 2×/week; high pulls, power cleans, and back squats; 3–6 sets of 4–6 reps; 80% 1RM. Kettlebell/TRT group: 2×/week; kettlebell swings, goblet squats; 3–6 sets of 4–6 reps; 16 kg kettlebell
				CMJ	Jump height (cm)		
Channell and Barfield [34]	27, WLT group (11), TRT group (10), CON group (6)	15.9 ± 1.2	Male, student athletes, from the high school football programme, limited resistance training experience	Power clean 1RM Back squat 1RM	Weight (kg)	8 weeks (prior 4 weeks of general strength only)	WLT group: 3×/week; power clean, hang clean, snatch pulls, snatch, push jerk and accessory strength; 3–5 sets of 5–10 reps; 60–75% 1RM. TRT group: 3×/week; Bench press, squat, dead lift, leg press, upper body press, back extensions, abdominals; 3–5 sets of 5–20 reps; 60–100% 1RM. CON group: did not participate in any off-season training
				CMJ	Height (cm)		
Hermassi et al. [39]	20, WLT group (10), CON group (10)	21.2 ± 0.7	Male, handball players, playing experience 10.1 ± 0.5 y	Snatch 1RM C&J 1RM	Weight (kg)	8 weeks	WLT group: 2×/week; a snatch from a squatting position, a bench-press, a half-squat, and a clean and jerk; 3–4 sets of 3–10 repetitions; 55–85% 1RM; 3 min. CON group: maintained standard in-season training regimen inclusive of TRT; physical conditioning 2×/week aimed at strength development
Pichardo et al. [59]	59, WLT group (31), TRT group (28)	WLT group 14.0 ± 0.5, 0.3 ± 0.6 from PHV, TRT group 13.9 ± 0.6, 0.1 ± 0.9 from PHV	Male, youth, < 1 year of resistance training experience, WLT naïve	IMTP	Peak force (N) Relative peak force (N/kg)	28 weeks	WLT group: WLT + field-based training; WLT 2×/week; 3-week introductory mesocycle using bodyweight exercises only, WL full lifts, WL derivatives and accessory strength; 1–5 sets of 5–12 reps. TRT group: FT + field-based training; WLT 2×/week; 3-week introductory mesocycle using bodyweight exercises only, squat lunge, upper body push and pull and core; 1–5 sets of 5–12 reps
				CMJ	Jump height (cm)		
				Horizontal jumps	Jump distance (m)		
				30-m sprint	Time (s)		
Hawkins et al. [60]	29, WLT group (9), TRT group (10), PLYO group (10)	21.5 ± 12.5	Male, ‘non-athlete’	Back squat 1RM	Weight (kg)	8 weeks	WLT GROUP: 3×/week, 60 min; WL full exercises, WL derivatives and accessory strength; 3 sets of 4–10 reps. TRT group: 3×/week, 60 min; squats, deadlifts and good mornings, lower body unilateral, upper body push and pull; 3 sets of 6–10 reps. PLYO group: 3×/week, 60 min; plyometrics; 3 sets of 6–15 reps
				SJ	Jump height (cm)		
				CMJ	Peak power (W) Eccentric utilisation ratio		
*Weightlifting training and plyometric training comparisons (n = 6)*							
Arabatzi et al. [61]	18, WLT group (9), PLYO group (9)	20.3 ± 2.0	Male, students of physical education, 1-year resistance training experience	SJ CMJ	Jump height (cm) Mean concentric power (W) Hip and knee displacement	8 weeks	WLT group: 3×/week; snatch from a squat position, high-pull, power clean, half-squat, and clean and jerk; 4 sets of 4–6 reps; 75–90% 1RM; 3-min rest. PLYO group: 3×/week; plyometrics, half-squat; 4 sets of 4–6 reps; 75–90% 1RM; 3-min rest. CON group: no training
Teo et al. [62]	26, WLT group (13), vertical jump/PLYO group (13)	24.2 ± 1.1	Male, minimum of 6 months’ resistance training experience	SJ CMJ DJ (40 cm)	Peak power (W)	6 weeks	WLT GROUP: 3×/week, 45 min; hang power clean and power snatch and half squat; 4–6 sets of 4–6 reps; 70% 1RM; 3–5-min rest. PLYO group: 3×/week, 45 min; plyometrics (jumps and drop jumps), half-squat;4–8 sets of 4–12 reps; 30–70% 1RM; 3–5 min rest
				5, 20-m sprint 505 (CODS)	Time (s)		
Hawkins et al. [60]	29, WLT group (9), TRT group (10), PLYO group (10)	21.5 ± 12.5	Male, ‘non-athlete’	Back squat 1RM	Weight (kg)	8 weeks	WLT group: 3×/week, 60 min; WL full exercises, WL derivatives and accessory strength; 3 sets of 4–10 reps. TRT group: 3×/week, 60 min; squats, deadlifts and good mornings, lower body unilateral, upper body push and pull; 3 sets of 6–10 reps. PLYO group: 3×/week, 60 min; plyometrics; 3 sets of 6–15 reps
				SJ	Jump height (cm)		
				CMJ	Peak power (W) Eccentric utilisation ratio		
Tricoli et al. [37]	21, WLT group (7), PLYO group (7), CON group (7)	22.0 ± 1.5	Male, limited WLT experience	½ Back squat 1RM	Weight (kg)	8 weeks	WLT group: 3×/week; WL full exercises, WL derivatives and accessory strength; 3–4 sets, 4–6 reps; 80–90% 1RM. PLYO group: 3×/week; plyometrics, half-squat; 4–10 reps, 4–6 reps; 80–90% 1RM. CON group: no training
				SJ CMJ	Height (cm)		
				10, 30-m sprint 4-m agility (CODS)	Time(s)		
Moore et al. [36]	15, WLT group (8), PLYO group (7)	20.2 ± 0.2	Male (10) and female (5), limited resistance training experience	Squat 4RM	Weight lifted (kg)	12 weeks	WLT group: 3×/week; hang clean and accessory strength; 3 sets, 6 reps; 85% 1RM. PLYO group: 3×/week; plyometrics; 1–3 sets, 10–30 reps
				CMJ	Jump height (cm)		
				25-m sprint	Time (s)		
Kaabi et al. [56]	45, WLT group (15), PLYO group (15), CON group (15)	16.6 ± 0.4	Male, ‘recreationally trained’ resistance training experience	Squat 1RM SJ CMJ	Height (cm)	8 weeks	5×/week table tennis technical sessions in addition to WLT or PLYO. WLT group: 2×/week; WL derivatives and accessory strength; 3–4 sets, 5–10 reps; 70–85% 1RM; 3–5-min rest. PLYO group: 2×/week; plyometrics; 3–4 sets, 5–10 reps; 70–85% 1RM; 3–5-min rest. CON group: 5×table tennis technical sessions/week with no TRT
				5-m sprint T test (CODS)	Time (s)		

*1RM* one repetition maximum, *C&J* clean and jerk, *CMJ* countermovement jump, *CODS* change of direction speed, *DJ* drop jump, *IMTP* isometric mid-thigh pull, *NCAA* National Collegiate Athletic Association, *PHV* peak height velocity, *PLYO* plyometric training, *RFD* rate of force development, *SJ* squat jump, *WL* weightlifting, *WLT* weightlifting training

The median age of the participants in the studies was 20.3 years (range 14–24). Four studies included youth participants, with the term youth referring to the period of life before adulthood and including individuals under 18 years of age [66]. Of these studies, only one included information on participants’ stage of maturation [59], using the maturity offset method to estimate maturity status [67]. The majority of studies were conducted with solely male participants (*n* = 12), with three of the studies including both male and female participants, and one study with female participants only.

The authors of four studies failed to provide information regarding the participants’ WLT experience prior to the intervention [40, 55, 57, 61]. Four studies included participants with no, or very limited, experience of WLT. The remaining eight studies included participants with limited resistance training experience (< 2 years); thus, it can be inferred that the participants in these specific studies were also inexperienced in weightlifting.

#### Weightlifting Intervention

The median duration of the WLT was 8 weeks (range 6–28). The majority of studies (*n* = 11) implemented WLT interventions lasting between 6 and 8 weeks, four studies employed a 10- to 15-week WLT intervention, and one study implemented a weightlifting intervention for 28 weeks’ duration. The median training frequency was three times per week (range 2–4). In seven of the studies, the training frequency was twice weekly and in one study, four times per week. All studies included a weightlifting intervention consisting of variations of the full weightlifting movements (snatch, clean and jerk), weightlifting derivatives (e.g. hang clean, hang snatch, power clean, clean pulls) and accessory strength exercises. One study used an intervention that included split-style weightlifting derivatives (hang split snatch, hang split clean, split jerk) only.

The authors of two studies failed to provide information on the training intensity prescription in relation to percentage of one repetition maximum (% 1RM) [33, 59]. Training intensity prescribed in the remaining studies ranged from 55 to 95% 1RM. Training volumes in the studies ranged from one to seven sets of 3–12 repetitions. Prescribed rest periods were 3–5 min across most of the studies (*n* = 9); however, this information was absent from a number of studies (*n* = 7) [33, 34, 36, 37, 58–60].

### Weightlifting Training Versus Control Group

**Fig. 2:**
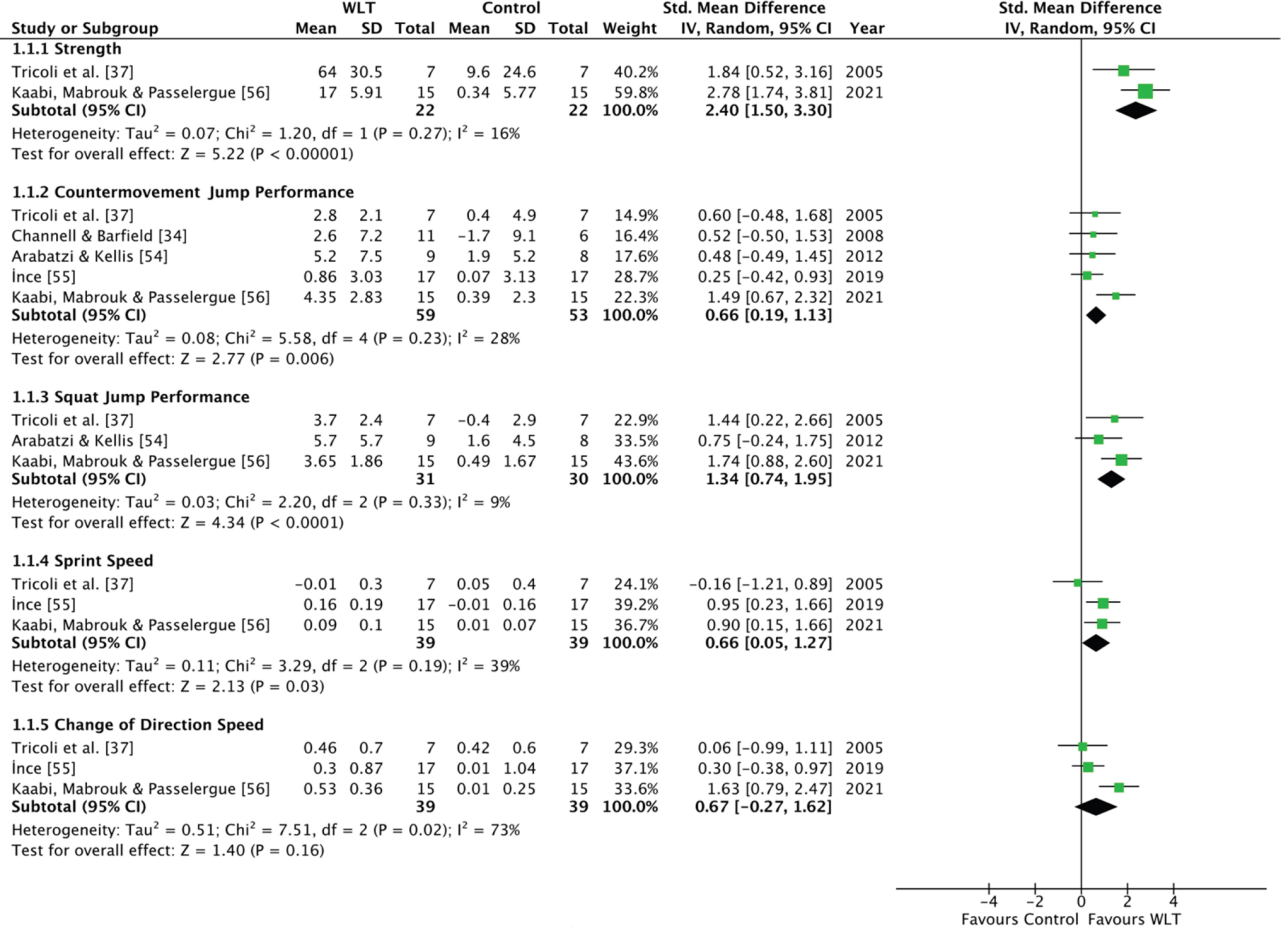
Forest plot for WLT group and CON group comparisons. *CON* control group, *Mean* pre–post intervention mean difference, *SD* pre-intervention standard deviation, *WLT* weightlifting training

Results are presented in Fig. 2. A large, significant effect in favour of WLT for improvements in strength (*p* < 0.001, *g* = 2.40; 95% CI 1.50–3.30) and SJ performance (*p* < 0.001, *g* = 1.34; 95% CI 0.74–1.95) was identified from the analysis of two and three studies, respectively. A moderate, significant effect in favour of WLT for improvements in countermovement jump (CMJ) performance (*p* = 0.006; *g* = 0.66; 95% CI 0.19–1.13) and sprint speed (*p* = 0.03, *g* = 0.66; 95% CI 0.05–1.27) was indicated in the analysis of five and three studies, respectively. A moderate, non-significant effect in favour of WLT for improvements in CODS (*p* = 0.16, *g* = 0.67; 95% CI − 0.27 to 1.62) was indicated in the analysis of three studies. High statistical heterogeneity was present in the CODS comparisons and the chi-square test for heterogeneity was significant (*I*^2^ = 73%; *p* = 0.020); however, all other variables only presented with small to medium levels of heterogeneity (Table 3). A large, predicated range of effects was evident across the variables (Table 3).

**Table 3:** Measures of heterogeneity across study comparisons

Assessment	*I*^2^	*χ*^2^ significance	Prediction interval
*Weightlifting group and control group comparisons*			
Strength	16%	0.270	
CMJ	28%	0.230	− 0.52 to 1.84
SJ	9%	0.330	− 3.19 to 5.87
Speed	39%	0.190	− 5.12 to 6.44
CODS	73%	0.020	− 10.30 to 11.64
*Weightlifting group and traditional resistance training comparisons*			
WL Performance	82%	0.000	− 3.86 to 6.58
Strength	67%	0.003	− 1.37 to 1.75
CMJ	89%	0.000	− 2.35 to 4.25
SJ	69%	0.010	− 2.14 to 2.86
Speed	90%	0.000	− 5.14 to 7.22
CODS	92%	0.360	
*Weightlifting training and plyometric training comparisons*			
Strength	77%	0.004	− 3.97 to 5.35
CMJ	0%	0.680	0.31
SJ	0%	0.790	0.34
Speed	0%	0.490	0.20
CODS	14%	0.310	− 3.80 to 4.14

*CMJ* countermovement jump, *CODS* change of direction speed, *SJ* squat jump, *WL* weightlifting

### Weightlifting Training Versus Traditional Resistance Training

**Fig. 3:**
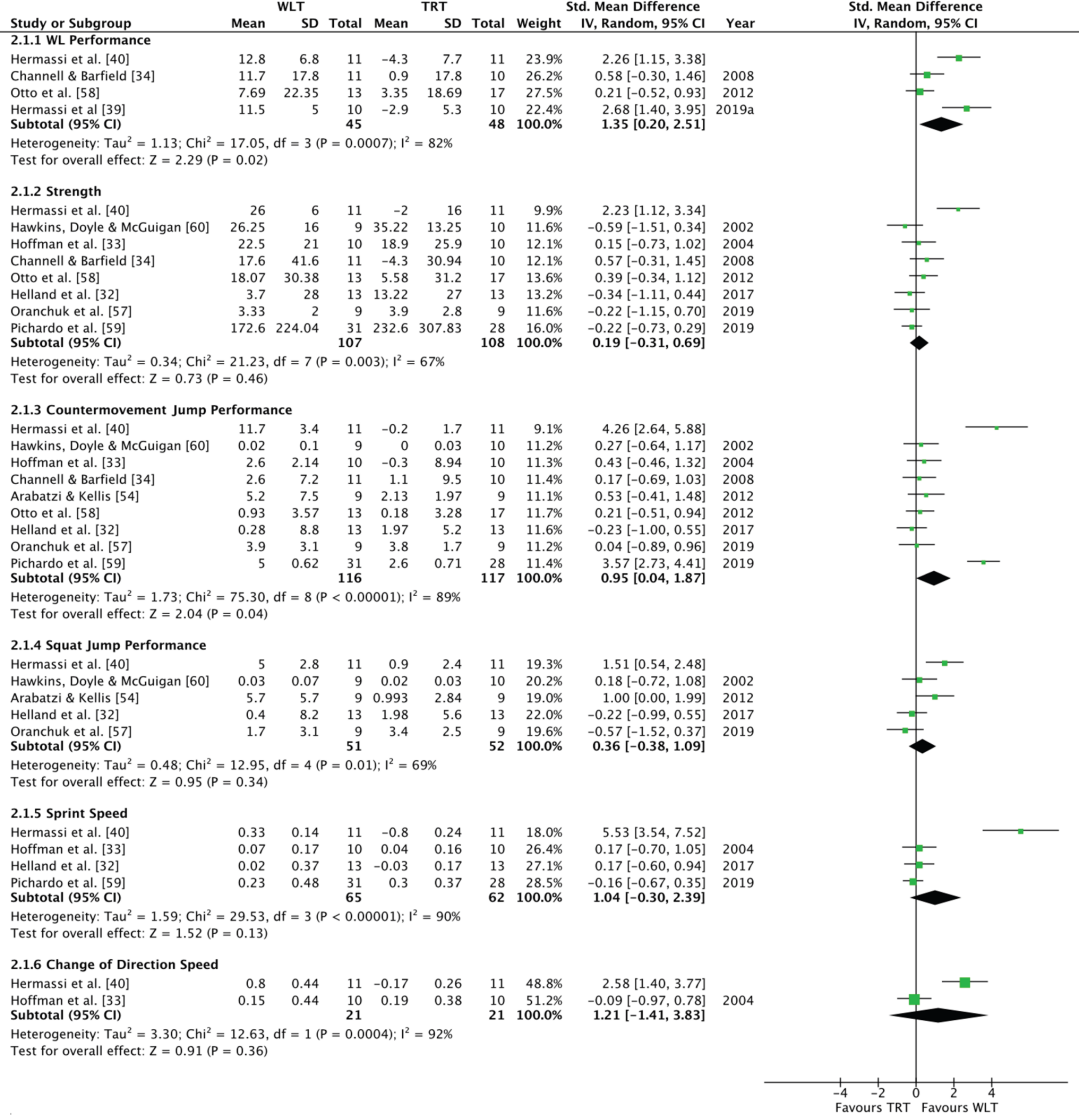
Forest plot for WLT group and TRT group comparisons. *Mean* pre–post intervention mean difference, *SD* pre-intervention standard deviation, *TRT* traditional resistance training, *WLT* weightlifting training

Combined data from four studies revealed a large, significant effect in favour of WLT for improvements in weightlifting performance (*p* = 0.02, *g* = 1.35; 95% CI 0.20–2.51) (Fig. 3). No effect was evident for improvements in strength (8 studies, *p* = 0.46, *g* = 0.19; 95% CI − 0.31 to 0.69) or SJ performance (5 studies, *p* = 0.34, *g* = 0.36; 95% CI − 0.38 to 1.09) (Fig. 3). A large, non-significant effect was found in favour of WLT for improvements in sprint speed (4 studies, *p* = 0.13, *g* = 1.04; 95% CI − 0.03 to 2.39) and CODS (2 studies, *p* = 0.36, *g* = 1.21; 95% CI − 1.41 to 3.83) (Fig. 3). A large, significant effect was found in favour of WLT for improvements in CMJ (9 studies, *p* = 0.00, *g* = 0.95; 95% CI 0.04–1.87) (Fig. 3). High statistical heterogeneity was present across all comparisons, and the chi-square test for heterogeneity was significant across all variables other than CODS (*I*^2^ = 67–92%; *p* < 0.010) (Table 3). A large, predicated range of effects was evident across the variables (Table 3).

### Weightlifting Training Versus Plyometric Training

**Fig. 4:**
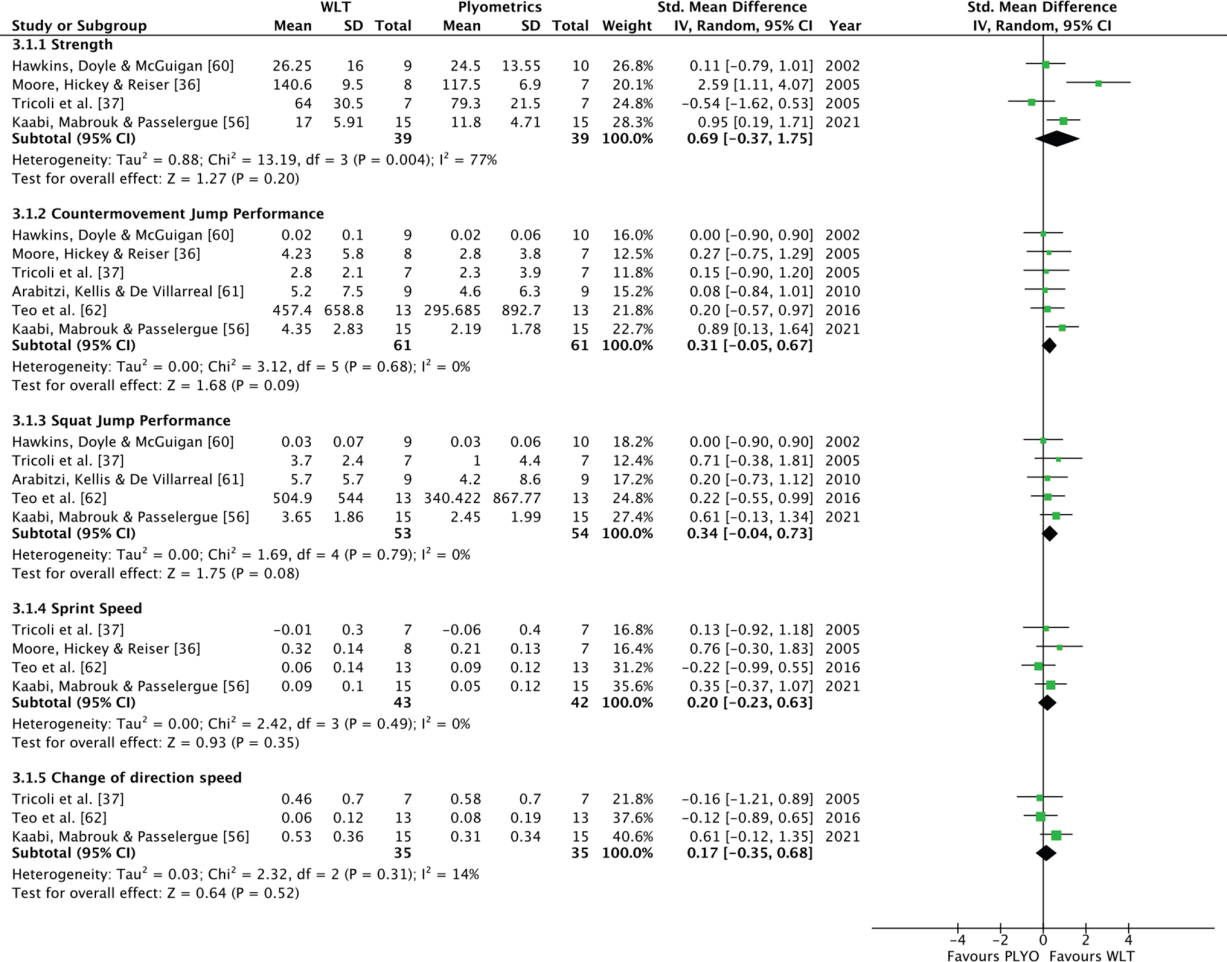
Forest plot for WLT group and PLYO group comparisons. *Mean* pre–post intervention mean difference, *PLYO* plyometric training, *SD* pre-intervention standard deviation, *WLT* weightlifting training

WLT and PLYO resulted in similar improvements in speed, power and strength as demonstrated by negligible to moderate, non-significant effects in favour of WLT for improvements in sprint speed (4 studies, *p* = 0.35, *g* = 0.20; 95% CI − 0.23 to 0.63), CODS (3 studies, *p* = 0.52, *g* = 0.17; 95% CI − 0.35 to 0.68), CMJ (6 studies, *p* = 0.09, *g* = 0.31; 95% CI − 0.05 to 0.67), SJ (5 studies, *p* = 0.08, *g* = 0.34; 95% CI − 0.04 to 0.73) and strength (4 studies, *p* = 0.20, *g* = 0.69; 95% CI − 0.37 to 1.75) (Fig. 4). High statistical heterogeneity was present in the strength comparisons and the chi-square test for heterogeneity was significant (*I*^2^ = 77%; *p* = 0.004); however, only small levels of heterogeneity were evident for the CMJ, SJ, speed and CODS comparisons (Table 3). A large, predicated range of effects was evident for strength and CODS, however CMJ, SJ and speed studies shared a common effect size (Table 3).

## Discussion

The study aimed to explore whether WLT resulted in greater improvements in measures of strength, power, speed and CODS compared with TRT, PLYO or CON. Findings from a limited number of studies suggested there are moderate to large benefits of WLT for improvements in strength, CMJ, SJ and speed performance when compared with no additional training beyond sports practice or typical physical activities. Whilst improvements in strength were found to be similar following both WLT and TRT, WLT may be superior for improvements in weightlifting performance (i.e. load lifted) and CMJ height, although high levels of heterogeneity suggest factors such as population characteristics or programme design may also influence these outcomes. Limited differences exist between WLT and PLYO for improvements in strength, jump, sprint speed and CODS performance. Cumulatively, these results underline the notion of training specificity; WLT is most effective for improving weightlifting performance, limited differences exist between WLT, TRT and PLYO for increasing strength, linear sprint speed and CODS, while WLT or PLYO is recommended to enhance jumping.

### Weightlifting Training Versus Control Group

Moderate to large effects favouring WLT across all variables in the WLT versus CON analysis indicate that WLT is more effective than no supplementary training for improving measures of strength, power and speed. These findings corroborate previous meta-analytical data that showed WLT could elicit moderate improvements in CMJ performance in comparison with CON group data [13]. Furthermore, based on the intervention characteristics of the studies included in the meta-analysis, three WLT sessions per week, for an 8-week period, is deemed a sufficient training dosage to elicit improvements in measures of strength, power and speed in athletes with limited weightlifting experience.

The large ES for improvements in strength and SJ performance, compared with moderate improvements in speed and CMJ performance, are likely due to the high similarities between the movement patterns and demands placed on the neuromuscular system in the squat, SJ and weightlifting movements [68]. Specifically, SJ performance is more dependent on concentric strength, whilst CMJ and speed performance are more dependent on utilisation of the stretch–shortening cycle (SSC) [69, 70]. This notion is supported by research that has shown SJ height to be the strongest correlate (*r* = 0.64) with weightlifting performance when compared with CMJ height and IMTP variables [71]. Cumulatively, these findings indicate that in athletes with limited weightlifting experience, WLT may predominantly elicit improvements in concentric force production, with the high power and propulsive force outputs typically exhibited in the weightlifting movements [17] appearing to principally transfer to improvements in strength and SJ performance.

Whilst moderate ES were observed in favour of WLT for improvements in CODS, these were found to be non-significant. Large improvements in strength and SJ performance, compared with only moderate improvements in CMJ and sprint speed, and non-significant, moderate improvements in CODS support the notion that in less experienced weightlifters, a delayed WLT effect might be present whereby a 6- to 8-week training duration may not be sufficient to translate newly developed strength properties into higher velocity tasks (e.g. CMJ and sprinting) often used to reflect athletic performance [72]. Furthermore, the lack of a significant improvement in CODS may be due to the multifactorial nature of CODS performance; physical qualities (measures of sprint speed and strength) have been found to explain only 57% of the variance associated with CODS performance [73]. Since physical qualities only partially underpin CODS performance, other task-specific technical factors (i.e. foot placement, posture and stride adjustment) should also be considered in training programmes aimed at improving CODS [74, 75]. Likewise, since the demands of CODS are multi-directional, the uni-directional nature of the weightlifting movements may have been accountable for low specificity to CODS gains. WLT may therefore not provide a training stimulus specific enough to improve the multi-directional and technical demands that underpin CODS performance.

### Weightlifting Training Versus Traditional Resistance Training

Interpretation of the findings may indicate that WLT is superior to TRT for stimulating improvements in weightlifting performance, which are likely attributable to the principle of training specificity [76, 77]. Weightlifting performance is not purely dependent on physical qualities such as strength and power, with technical factors (e.g. posture, weight distribution, bar position) also influencing performance [78–80]. The nature of WLT clearly provides opportunities to develop and refine weightlifting technique and movement skill acquisition [81], thus increasing the load an athlete is able to lift in the weightlifting movements (i.e. clean and snatch), which would not be experienced from TRT alone. Furthermore, the weightlifting exercises combine high force and high velocity movements, requiring continuous acceleration throughout the entire movement [82]. In comparison, TRT usually utilises heavier loads and has a natural deceleration component to the end of the lift [83]. Therefore, in comparison to WLT, TRT may require expressions of force at slower velocities [84–86]. Since adaptations are dependent on the particular training stress applied, it is likely that the lack of exposure to high velocity movements and dissimilar accentuated regions of force in TRT alone do not provide an adequate stimulus to elicit improvements in weightlifting performance [76].

There was limited difference in the magnitude of strength gains made from WLT and TRT, as indicated by small, non-significant ES in favour of WLT. Similarities in strength gains may be due to correspondences in the training stimuli since all of the WLT interventions incorporated accessory strength work, whereby exercises similar to those included in the TRT were also performed (e.g. back squats, bench press and lunges). Therefore, because of the inclusion of accessory strength work in the WLT programmes, it is not possible to determine with accuracy the sole contribution of the WLT on strength gains. Additionally, the principle of training specificity may explain why similar improvements were evident from TRT and WLT in force-dominant assessments, since both training methods include exercises that demand high force expressions [87, 88]. Furthermore, all of the TRT and WLT programmes included in the WLT versus TRT analysis included the squat exercise, providing a further, test-specific training stimulus.

When considering study outliers, data from Hermassi et al. [40] show large, significant improvements in strength, SJ, CMJ, sprint speed and CODS, with larger ES in favour of WLT in comparison with the other studies. During this intervention, participants completed a total of eight training sessions per week for a 12-week training period, which was a greater training dosage than the other studies included in the meta-analysis and may have led to greater changes from the WLT intervention. The increased training exposure would have allowed for a longer period for training adaptations to manifest [89, 90], ultimately leading to greater training improvements in strength, power and speed. The limited improvements in the TRT group may have also been responsible for the greater ES found in favour of WLT, however limited information on the TRT prescription employed in the study prevent any further exploration [40]. Pichardo et al. [59] reported large ES for CMJ performance in favour of WLT in comparison with the other studies included in the meta-analysis. The training intervention duration employed in the study was 28 weeks [59], which may have resulted in more pronounced improvements in measures of power compared with the other studies included in the WLT versus TRT analysis that implemented shorter training durations (median training duration: 8 weeks, range 6–28) [90]. In addition, the participants in the study were adolescent boys, which may have heightened the training response, as adolescents may be capable of greater absolute gains from training in comparison with adults, owing to concomitant growth and maturity-related adaptations (e.g. morphological changes and neural adaptations) [91].

It is likely that neural mechanisms are primarily responsible for high force outputs [92] and improvements in rate coding, motor unit recruitment and motor unit synchronisation have been shown to typically occur as a result of high load, or high velocity training [93, 94]. WLT provides both a high load and high velocity training stimulus in comparison with TRT exercises, which are performed as slower speeds [87]. Therefore, TRT may best elicit adaptations that underpin maximum force production, whilst WLT may also elicit improvements underpinning the velocity components. Furthermore, research suggests that, providing the training duration is sufficient, increases in muscle cross-sectional area (mCSA) may be more prevalent from TRT compared with WLT [35]. These adaptations may be attributed to the slower movements, increased time under tension and accumulation of metabolic fatigue in TRT exercises that is not typically apparent in WLT because of differences in the loading parameters [95]. For example, the technical demands of the weightlifting movements tend to deter high volumes of training at high loads. Limited changes in mCSA from WLT compared with TRT, but similar improvements in strength, may infer that neural mechanisms and changes in co-ordination were responsible for some of the WLT improvements. In support of this, previous researchers have suggested that WLT improves power performance via a constant co-activation index, in comparison with TRT which resulted in an increase in co-activation index [54]. These findings may imply that WLT may improve coordination of antagonistic muscle groups. However, future research exploring muscle activation and changes in muscle architecture after WLT interventions is needed to confirm this speculative notion.

Whilst WLT and TRT were both effective at improving strength, the results suggest that WLT may offer additional advantages over TRT for improvements in CMJ performance; with large, significant ES in favour of WLT. Furthermore, large ES in favour of WLT were also evident for improvements in sprint speed and CODS, albeit these were non-significant. Similar improvements in strength but greater improvements in CMJ indicate that the combination of high force and high velocity indicative of weightlifting movements may result in adaptations in a greater range of measures across the force–velocity curve in comparison with TRT [5, 20]. Previous researchers have presented data that provides evidence that the hang power clean, sprinting and jumping performance are significantly correlated, suggesting that these performance variables are underpinned by similar underlying strength qualities [96]. It could be speculated that in comparison with TRT alone, the greater movement complexity required for the weightlifting movements may result in different neural adaptations (e.g. motor unit recruitment, rate coding) in comparison with TRT, which further facilitate adaptations across a broader range of physical capacities after a sufficient training duration [19]. Furthermore, the continual acceleration required in the weightlifting movements [82] is similar to that of jumping performance, therefore the movement characteristics in weightlifting may also elicit superior improvements in athletes’ speed–strength qualities [5]. Overall, these findings support the notion that if the training goal is to improve strength, power and speed, such is the case with many team sport athletes, WLT may be a more efficient means of training for a broad spectrum of physical qualities in comparison with TRT alone.

### Weightlifting Training Versus Plyometric Training

Findings suggest that WLT may elicit similar improvements in CMJ, SJ, speed and CODS when compared with PLYO, as demonstrated by non-significant, negligible-to-small ES. Similar to weightlifting movements, plyometric exercises such as jumping, hopping and bounding are performed with maximum acceleration throughout triple extension at hip, knee and ankle [86, 97]. These explosive movements produce adaptations transferable to a range of sporting movements [5], which may suggest why limited differences between the two training methods were found for sprinting and jumping performance. Similar magnitude of effects have been reported in extant meta-analyses when comparing the effects of WLT and PLYO on improvements in CMJ (ES = 0.15 [14], ES = 0.11 [13]). However, while improvements in jumping and sprinting from WLT and PLYO were found to be similar in the current study, it has been suggested that the mechanisms behind these changes may differ [13]. Although speculative in nature, improvements in sprint speed and jump performance from WLT and PLYO may be due to adaptations related to motor learning, coordination and motor unit recruitment [54, 98]. However, in addition to this, improvements following PLYO may also be dependent on changes in the mechanical properties of the muscle–tendon complex [99, 100] with higher levels of stiffness facilitating greater amounts of stored and reused elastic energy [101]. Differences in the adaptation mechanisms suggest a synergistic effect might be evident if both WLT and PLYO were included in a training programme [35, 91]. It is important to note that the sprint distances in the studies used in this meta-analysis comparison were 20–30 m. Similar training effects between WLT and PLYO may not be evident when sprinting over a longer distance (40–100 m). Previous researchers have suggested performance of the initial acceleration (0–10 m) is affected mainly by concentric action and power performance [102], whereas the later phase of maximal velocity is also affected by muscle–tendon stiffness [103]. Therefore, greater transfer could result from PLYO compared with WLT when sprinting over a longer distance due to greater ability to utilise elastic energy, hence less deceleration over the latter phase of the sprint.

In comparison with WLT movements, PLYO typically consists of high velocity movements performed without external load [104]. In accordance with the principle of training specificity [76], heightened improvements in a high-load, strength-dominant movement such as a 1RM squat may therefore be expected as a result of the training demands of WLT in comparison with PLYO. In contrast, findings from the current review suggest in athletes with limited plyometric and resistance-training experience (< 2 years), WLT and PLYO may elicit similar improvements in strength.

## Limitations

The interpretation of findings should recognise the high heterogeneity across the studies included, particularly in the WLT and TRT comparisons. Findings of high heterogeneity may be due to variations in data collection protocols and training interventions across the studies. The high heterogeneity (*I*^2^ > 75%) along with small sample sizes may have been responsible for instances of non-significant differences, despite the existence of large ES [50]. In addition, results from the sensitivity analysis in the WLT and TRT comparison do not complement the conclusions of the primary analysis for all main outcome parameters, thus suggesting the quality of the studies may have influenced the results. The majority of studies had small sample sizes, which may be a result of the demands of delivering a large-scale WLT study. WLT typically requires a higher coach-to-athlete ratio than sports coaching sessions, therefore conducting a large-scale WLT may present additional, logistical challenges. Due to the small number of studies meeting the inclusion criteria, there were no limitations on study population, and participant age, maturity or sex may indeed moderate the training response. Furthermore, there was a lack of exclusivity of training exercises in a number of the study interventions (e.g. accessory strength work included in the WLT interventions; kettlebell exercises and ballistic exercises included within the TRT interventions). Therefore, it is not possible to determine the independent contributions of the weightlifting, plyometric and resistance training exercises to the overall training effects, with the current study comparing these training methods in more broad terms.

The authors also recognise the risk of systematic and random errors associated with a single reviewer approach to screening [105]. However, to reduce these risks, articles were dually screened by the reviewer and all study exclusion and data extraction was verified by a second reviewer. Furthermore, whilst a single reviewer approach may result in wider confidence intervals, it is likely that the direction of the findings from the meta-analysis would not differ [106], thereby allowing a valid comparison between training interventions.

## Future Research

Analysis of the included studies indicates that there is a lack of randomised, controlled WLT studies, particularly involving youth participants, female participants and intermediate or advanced level weightlifters. Furthermore, the majority of included studies implemented short-term training interventions (6–8 weeks). Research indicates TRT duration has a significant effect on improvements in muscle strength [107]. Notably, whilst the greatest improvements in strength for untrained athletes can be experienced in the first 3 months of training, research indicates a trend toward slower rates of progression with training experience [108, 109]. There is a need for future research to implement long-term WLT interventions to explore how improvements and the mechanisms of improvement from WLT may change over longer training durations. Furthermore, given the effectiveness of WLT, TRT and PLYO evidenced in the current review, future research should investigate the means by which these training methods can be effectively combined in a periodised plan.

## Conclusion

The current study revealed that WLT is an effective training method to improve strength, CMJ, SJ and sprint speed performance. When compared with alternative training modalities, WLT may elicit additional benefits above that of TRT alone, resulting in greater improvements in weightlifting and CMJ performance. WLT and PLYO may result in similar improvements in strength, jump performance and speed. Overall, these findings support the notion that if the training goal is to improve strength, power and speed, the inclusion of weightlifting exercises within phases of the training cycle may be advantageous to target goal-specific adaptations while also promoting the development of a well-rounded athlete.

## Supplementary Information

Below is the link to the electronic supplementary material.

Supplementary file1 (DOCX 3258 kb)

## Declarations

### Data availability statement

All data generated or analysed during this study are included in this published article (and its supplementary information files).
