# Supplementary material for: Comparison of Weightlifting, Traditional Resistance Training and Plyometrics on Strength, Power and Speed: A Systematic Review with Meta-Analysis
Source: Sports Med. 2022 Jan 13;52(7):1533–54. doi: 10.1007/s40279-021-01627-2 (PMC9213388; doi:10.1007/s40279-021-01627-2)
Supplement: Supplementary file 1 — Supplementary file1 (DOCX 3258 kb) [file 40279_2021_1627_MOESM1_ESM.docx]

APPENDICES
**Appendix 1.** Database Search Terms

**1) MEDLINE (via Ovid)**

(olympic or snatch* or power-clean* or hang-clean* or clean-and-jerk or jerk* or high-pull* or weightlift*) AND (training or intervention).mp.

=1519

**2) SPORTDiscus (EBSCOhost)**

TI (olympic or snatch* or power-clean* or hang-clean* or clean-and-jerk or jerk* or high-pull* or weightlift*) ) OR AB ( olympic or snatch* or power-clean* or hang-clean* or clean-and-jerk or jerk* or high-pull* or weightlift*) ) AND TI ( training OR intervention ) OR AB ( training OR intervention)

=1906

**3) PUBMED**

(("olympic"[Title/Abstract] OR "snatch*"[Title/Abstract] OR "power clean*"[Title/Abstract] OR "hang clean*"[Title/Abstract] OR ("clean-and-jerk"[Title/Abstract] OR "jerk*"[Title/Abstract]) OR "high pull*"[Title/Abstract] OR "weightlift*"[Title/Abstract]) AND ("training"[Title/Abstract] OR "intervention"[Title/Abstract])) AND (journalarticle[Filter])

=1573

**4) SCOPUS**

( TITLE-ABS-KEY ( ( olympic OR snatch* OR power-clean* OR hang-clean* OR clean-and-jerk OR jerk* OR high-pull* OR weightlift* ) ) ) AND ( TITLE-ABS-KEY ( ( training OR intervention ) ) ) AND ( LIMIT-TO ( DOCTYPE , "ar" ) )

=2649

**Appendix 2.** Categorization of strength and power assessments.

| **Category** | **Test** | **Measure** |
| --- | --- | --- |
| Weightlifting Performance | Clean & jerk 1RM | Weight (kg) |
|  | Power clean 1RM | Weight (kg) |
| Strength | Half-squat 1RM | Weight (kg) |
|  | Back squat 1RM | Weight (kg) |
|  | Smith Machine Squat 4RM | Weight (kg) |
|  | IMTP | Absolute peak force (N)  Relative peak force (N/kg) |
| Countermovement Jump Performance | CMJ | Jump height (cm) |
| Squat Jump Performance | SJ | Jump height (cm) |
| Sprint Speed | 5m sprint  20m sprint | Time (s)  Time (s) |
|  | 25m sprint | Time (s) |
|  | 30m sprint | Time (s) |
|  | 40-yard sprint | Time (s) |
| Change of Direction Speed | 505 | Time (s) |
|  | Agility Test (4m Box) | Time (s) |
|  | T-test | Time (s) |

**Appendix 3.** Tool for the assEssment of Study qualiTy and reporting in EXercise (TESTEX) Scale

| **Item** | **Question** | **Additional Information** | **Scoring** |
| --- | --- | --- | --- |
| **Study Quality** | | | |
| 1 | Eligibility criteria specified | Eligibility criteria should be specified and fulfilled, and specific diagnostic test values should be provided for all participants. | Yes (1) /No (0) |
| 2 | Allocation concealment | It should be stated if group allocation was concealed; meaning if a patient was eligible for inclusion in the trial was unaware (when this decision was made) of which group the patient would be allocated to. Yes if group allocation was concealed from patients eligible for inclusion in the trial (e.g. consent should be given before randomization). | Yes (1) /No (0) |
| 3 | Randomization specified | A description of the method used to allocate patients into treatment groups should be provided. Yes, if methods are described and they are truly random e.g., coin-tossing, sequence of randomly generated numbers. | Yes (1) /No (0) |
| 4 | Groups similar at baseline | Baseline data of all participants who were randomized should be presented. There should be no significant difference in the measure of the severity of the treated condition between treatment groups. 1 Point – if baseline data are separated by group allocation, presented and no differences are apparent. | Yes (1) /No (0) |
| 5 | Blinding of assessor for at least one key outcome | It is not always possible to blind patients and/or therapists; however, blinding of assessors is reasonable. If assessors of primary outcome measures are blinded to the intervention allocation of the patients, this should be stated clearly. | Yes (1) /No (0) |
| **Study Reporting** | | | |
| 6 | Assessment of outcome measures | The percentage of patients completing the study in both groups should be reported. 1 Point if adherence>85%, 1 point if adverse events are reported and 1 point if exercise attendance is reported. | Yes (3) /No (0) |
| 7 | Intention-to-treat analysis | When a patient withdraws, this analysis is conducted by using either the last value obtained for each of the outcome measures as a post-intervention value, or by using the baseline value as a post value. This analysis should be added to the data of those that did complete the study and an analysis conducted. 1 point for no withdrawal. | Yes (2) /No (0) |
| 8 | Between-group statistical comparisons reported | Comparison of exercise vs. comparator (control) group for the primary and at least one secondary outcome should be performed. 1 Point if between-group statistical comparisons are reported for the primary outcome measure of interest, 1-point if f between-group statistical comparisons are reported for at least one secondary outcome measure. | Yes (1) /No (0) |
| 9 | Point measures and measures of variability for all reported outcome measures | Point estimates should be provided for all outcomes, otherwise this could be deemed selective outcome reporting. | Yes (1) /No (0) |
| 10 | Activity monitoring in control groups | Between-group differences may be diluted if control patients crossover to intervention. As many as one third of patients do this, so some measure e.g. exercise diary or activity monitoring should be supplied so this effect can be measured and quantified. 1 point if control patients are asked to report their levels of physical activity and data are presented. | Yes (1) /No (0) |
| 11 | Relative exercise intensity remained constant | Exercise intensity is considered by many to be the best stimulus for adaptation. Once patients begin an exercise programme at a set intensity they will begin to adapt. Throughout the study duration the relative intensity will fall in those that do adapt. Therefore, periodic assessment of exercise capacity should be conducted and the intensity titrated up (or in those that lose fitness, titrated down) so that exercise intensity remains constant. 1 point where attempt is made to keep relative intensity constant/ absolute intensity progressive. | Yes (1) /No (0) |
| 12 | Exercise volume and energy expenditure reported | Exercise parameters; session and programme duration, session frequency, exercise training intensity and modality should be clearly reported. | Yes (1) /No (0) |

**Appendix 4.** Funnel plots for all main analyses.


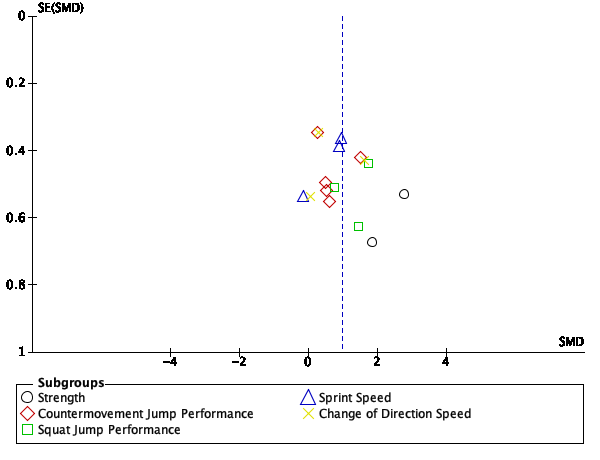

**Figure 1.** WLT vs CON


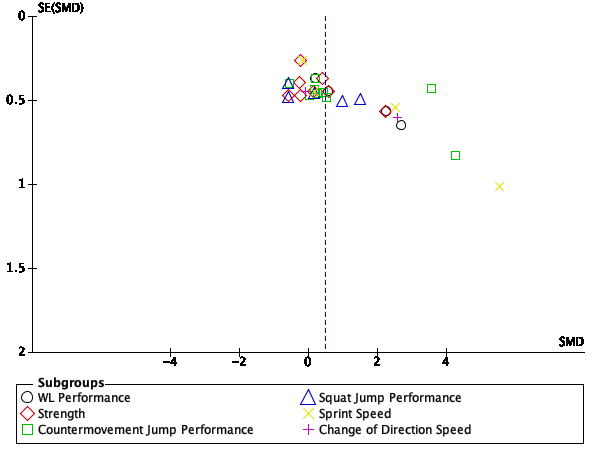


**Figure 2.** WLT vs TRT


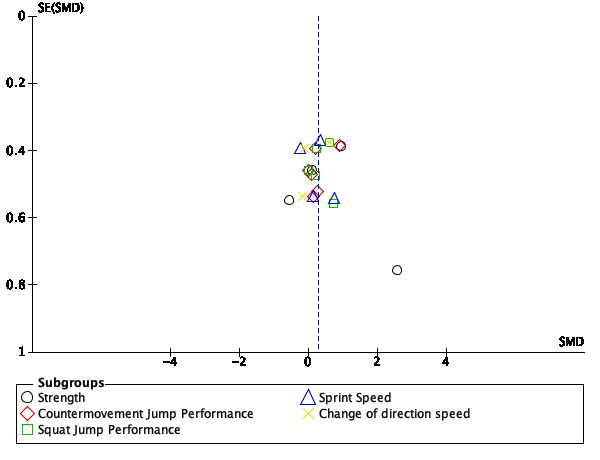

**Figure 3.** WLT vs PLYO

**Appendix 5.** Sensitivity analysis with exclusion of fair quality studies
WLT = Weightlifting training, TRT = Traditional Resistance Training, PLYO = Plyometric Training, WL = Weightlifting, CMJ = Countermovement jump, SJ = Squat jump, CODS = Change of direction speed.

| **Outcome** | **Studies (N)** | **ES (95% CI)** | **P Value** |
| --- | --- | --- | --- |
| **WLT vs CON** | | | |
| Strength | 2 | 2.40 (1.50 – 3.16) | 0.000 |
| CMJ | 3 | 0.92 (0.25 – 1.59) | 0.007 |
| SJ | 3 | 1.34 (0.74 – 1.95) | 0.000 |
| Sprint Speed | 2 | 0.44 (-0.60 – 1.47) | 0.41 |
| CODS | 2 | 0.88 (-0.67 – 2.42) | 0.26 |
| **WLT vs TRT** | | | |
| WL Performance | 4 | 1.35 (0.20 – 2.51) | 0.02 |
| Strength | 4 | -0.29 (-0.64 – 0.07) | 0.12 |
| CMJ | 5 | 0.77 (-0.72 – 2.26) | 0.31 |
| SJ | 4 | -0.02 (-0.73 – 0.69) | 0.95 |
| Sprint Speed | 2 | 1.14 (-1.49 – 3.77) | 0.40 |
| CODS | 2 | 1.21 (-1.41 – 3.83) | 0.36 |
| **WLT vs PLYO** | | | |
| Strength | 3 | 0.24 (-0.61 – 1.09) | 0.58 |
| CMJ | 4 | 0.36 (-0.06 – 0.79) | 0.09 |
| SJ | 4 | 0.37 (-0.05 – 0.80) | 0.08 |
| Sprint Speed | 3 | 0.09 (-0.38 – 0.56) | 0.70 |
| CODS | 3 | 0.17 (-0.35 – 0.68) | 0.52 |
